# Chronic Acquired Demyelinating Polyneuropathy following Renal Transplantation

**DOI:** 10.1155/2013/360454

**Published:** 2013-12-02

**Authors:** D. S. Younger, Stuart Orsher

**Affiliations:** ^1^Department of Neurology, NYU School of Medicine, New York University Langone Medical Center, 550 First Avenue, New York, NY 10016, USA; ^2^Department of Medicine, Lenox Hill Hospital, New York, NY 10021, USA; ^3^Section of Neurology, Lenox Hill Hospital, New York, NY 10021, USA

## Abstract

The clinical, laboratory, and treatment findings of a patient with chronic acquired demyelinating polyneuropathy (CADP) in association with renal transplantation are described. Like the present case, many such patients have been described under the rubric of chronic inflammatory demyelinating polyradiculoneuropathy (CIDP).

## 1. Introduction

The chronic acquired demyelinating polyneuropathies (CADP) are treatable heterogeneous disorders. Electrodiagnosis, which distinguishes primary demyelination from primary axonal neuropathy, achieves 95% sensitivity in CIDP [1]. Numerous conditions have been associated with CADP, among them solid organ transplantation [2]. Only ten heterogeneous patients [3] with preexisting axonal polyneuropathy met definite demyelinating electrodiagnostic criteria after solid organ transplantation [2]. We present the detailed clinical findings of another patient with progressive demyelinating neuropathy suggested by serial electrodiagnostic studies, diagnostic features on cutaneous nerve biopsy, and a positive response to intravenous immune globulin (IVIg), a preliminary report of which has been published [4].

## 2. Patient Report

**Figure 1:**
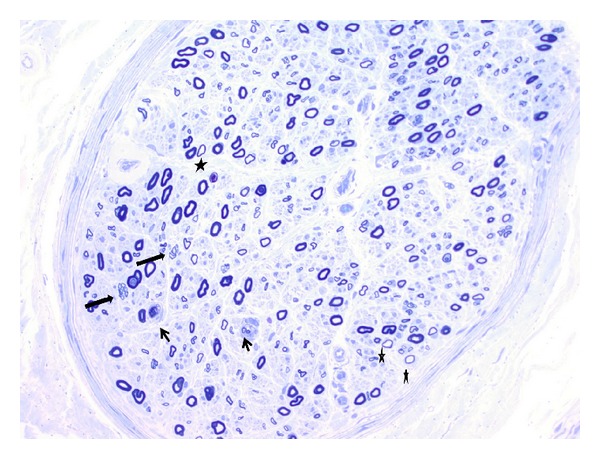
Semithin epoxy transverse section of sural nerve stained with toluidine blue. A transverse epoxy resin section shows mild axonal loss. Occasionally, clusters of small axons, suggesting axonal regeneration, are seen (long arrows). Thinly myelinated fibers are relatively often present (asterisks). There are a few macrophages in the section, engulfing the cell debris (short arrows) (×400).

A previously healthy 74-year-old man with solitary kidney developed renal insufficiency in 2008 prompting hemodialysis and eventual donor transplantation in 2009. Afterward, he was maintained on tacrolimus. This was followed shortly afterward by glycemic intolerance managed with diet. In 2011, he noted numbness, tingling sensation, and gait instability. Neurological examination showed tandem gait imbalance and symmetrical stocking vibratory and cold temperature sensory loss to below the knees, with slight weakness of the tibialis anterior muscles versus firm resistance that was graded Medical Research Council (MRC) 4/5 bilaterally. There was generalized hyporeflexia with intact limb strength, cognition, and cranial nerve function. Electrodiagnostic studies (Table 1) showed low amplitude compound muscle action potentials (CMAP), slow motor nerve conduction velocities, prolonged distal motor latencies, prolonged or absent F wave latencies, and abnormal temporal dispersion without active or chronic spontaneous activity at rest on concentric needle electromyography (EMG). Epidermal nerve fiber (ENF) studies of the left calf and thigh showed significantly low ENF density: distal leg 0.06 and thigh 5.4 ENF/mm (5th percentile reference values, resp., 5.0 and 8.0 ENF/mm 9) without histologic abnormalities or amyloid deposition. The serum creatine kinase was 172 U/L (normal < 150). Left sural nerve biopsy showed chronic and active peripheral neuropathy with primary demyelinating features and mild secondary axonal loss evidenced on teased nerve fiber studies in which 27 (54%) showed segmental remyelination, 23 (46%) were normal in configuration, and none showed Wallerian degeneration. Semithin epoxy resin sections of nerve (Figure 1) showed small clusters of regenerating axons, thinly myelinated fibers surrounded by Schwann cell processes, macrophage (CD68 positive) associated engulfment of myelin/axon debris, endoneurial mononuclear cell infiltration, and variation between fascicles. Soleus muscle biopsy showed severe myofiber atrophy consistent with a neurogenic abnormality. Treatment initiated with 0.4 g/kg of IVIg therapy twice monthly for one month, followed by 2 additional monthly courses, was associated with objective improvements in gait, sensation, and strength and demonstrable benefit on electrodiagnostic studies.

## 3. Discussion

The recommended strategy to confirm the electrodiagnosis of chronic inflammatory acquired demyelinating polyneuropathy is to increase sensitivity by examining more than four motor nerves including those on one side with the option of studying nerves on the opposite side of the body, adding proximal sites of stimulation, repeating the study at a later time if diagnostic criteria are not yet met, and providing supportive histopathologic evidence in a cutaneous nerve biopsy [2] as was accomplished in the present patient. The postulated causes of solid organ transplantation-associated progressive demyelinating disease include the triggering effects of immunosuppressive agents, preexisting nerve damage, and the surgical procedure itself. Despite the obvious challenge in establishing the electrodiagnostic diagnosis of demyelination in individual patients [3], the estimated 100-fold increase in the prevalence of the disorder in association with solid organ transplantation [2] makes it conceivable that CADP may be underestimated in patients undergoing renal transplantation.

## Figures and Tables

**Table 1:** Nerve conduction findings in a patient with progressive demyelinating neuropathy after renal transplantation.

	Motor nerve conductions								Sensory nerve conductions					
	CMAP (mV)		DL (ms)		Velocity (m/s)		F-response (ms)		SNAP (*µ*V)		DL (ms)		Velocity (m/s)	
Nerve/study 1	1	2	1	2	1	2	1	2	1	2	1	2	1	2
R. fibular	3.8	4.1	4.4	4.9	37	39	NR	65						
R. tibial	1.0	3.3	7.3	6.6	31	33	76	69						
R. sural									NR	3.0	—	3.5	—	28
R. sup. fibular									NR	NR				
L. fibular	1.5	3.1	6.9	5.4	37	42	NR	63						
L. sural									NR	NR				
L. sup. fibular	1.7	4.0	7.6	6.3	32	36	73	61						

1: study 1, November 2011; 2: study 2, June 2012. Abbreviations: L.: left; R.: right; CMAP: compound muscle action potential; DL: distal latency; mV: millivolt; ms: milliseconds; m/s: meters per second; SNAP: sensory nerve action potential; NR: no response; sup.: superficial.
